# Resveratrol represses YKL-40 expression in human glioma U87 cells

**DOI:** 10.1186/1471-2407-10-593

**Published:** 2010-10-28

**Authors:** Wei Zhang, Koji Murao, Xiang Zhang, Kensuke Matsumoto, Suwarni Diah, Masaki Okada, Keisuke Miyake, Nobuyuki Kawai, Zhou Fei, Takashi Tamiya

**Affiliations:** 1 Department of Neurosurgery, Xijing Hospital, The Fourth Military Medical University, 127 West Changle Road, Xi'an, Shaanxi Province, 710032, People's Republic of China; 2 Department of Neurological Surgery, Faculty of Medicine, Kagawa University, 1750-1 Ikenobe, Miki-cho, Kida-gun, Kagawa 761-0793, Japan; 3 Division of Endocrinology and Metabolism, Department of Internal Medicine, Faculty of Medicine, Kagawa University, 1750-1 Ikenobe, Miki-cho, Kida-gun, Kagawa 761-0793, Japan; 4 Department of Pharmacology, Faculty of Medicine, Kagawa University, 1750-1 Ikenobe, Miki-cho, Kida-gun, Kagawa 761-0793, Japan

## Abstract

**Background:**

Glioblastoma multiforme (GBM) is the most malignant intracranial tumour that develops in both adults and children. Microarray gene analyses have confirmed that the human YKL-40 gene is one of the most over-expressed genes in these tumours but not in normal brain tissue. Clinical studies have shown that serum YKL-40 levels are positively correlated with tumour burden in addition to being an independent prognostic factor of a short relapse-free interval as well as short overall survival in patients with various cancers. Our previous study revealed that YKL-40 was closely correlated with the pathological grades of human primary astrocytomas and played a crucial role in glioma cell proliferation. Hence, YKL-40 could be an attractive target in the design of anti-cancer therapies.

**Methods:**

Cell viability and invasion assays were performed to detect the cell proliferation and invasive ability of U87 cells induced by resveratrol (3, 5, 4'-trihydroxystilbene; Res) or YKL-40 small-interfering RNAs (siRNAs). In addition, the luciferase assay, real-time RT-PCR, western blotting, and ELISA were used to measure YKL-40 promoter activity, mRNA, and protein expression, respectively. The expressions of phosphor-ERK1/2 and ERK1/2 were determined by western blotting.

**Results:**

Res inhibited U87 cell proliferation and invasion *in vitro *and repressed YKL-40 in U87 cells by decreasing the activity of its promoter and reducing mRNA transcription and protein expression *in vitro*. YKL-40 siRNA treatment also impaired the invasiveness of U87 cells. When U87 cells were cultured with 20 μM PD98059 (an ERK1/2 inhibitor) alone, with 20 μM PD98059 and 100 μM Res, or with 100 μM Res alone for 48 h, YKL-40 protein expression decreased most significantly in the Res-treated group. PD98059 partially reversed the decrease of YKL-40 protein expression induced by Res. Furthermore, phosphor-ERK1/2 expression was reduced by Res treatment in a time-dependent manner.

**Conclusions:**

We demonstrated for the first time that Res represses YKL-40 expression *in vitro*; in addition, the ERK1/2 pathway is involved in this repression. This finding could extend the prospective use of Res in glioma research and enlarge the armamentarium for treating gliomas.

## Background

Malignant gliomas are the most common type of primary malignant brain tumours and are almost always lethal in both adults and children despite the existence of therapies, which include surgery, radiotherapy, and chemotherapy. Patients with GBM have an average survival time of 12 to 18 months from initial diagnosis and 6 to 9 months after recurrence [1,2].

In recent years, literature reviews of patients with various types of cancer (breast, colorectal, ovarian, kidney, prostate, small-cell lung, melanoma, and glioma) suggest that the serum YKL-40 level detected with the enzyme-linked immunosorbent assay (ELISA) could be a promising predictor of tumour burden and an independent prognostic variable of a short relapse-free interval as well as short overall survival [3-12]. More importantly, an elevated plasma YKL-40 level could predict an increased risk of gastrointestinal cancer after diagnosis of any other cancer [13].

Over a decade ago, YKL-40 was identified *in vitro *as a secreted glycoprotein in the culture medium of the human osteosarcoma cell line MG63 [14]. It contains an open reading frame of 383 amino acids and has a molecular weight of 40,476 kDa [15]. The gene encoding YKL-40 is located on chromosome 1q31-q32. The Sp1-family transcription factors predominantly regulate YKL-40 promoter activity during human macrophage differentiation [16]. Although the biological function of YKL-40 in cancer remains largely unclear, studies have suggested that YKL-40 may play a positive role in cancer cell survival and differentiation, protection against apoptosis, angiogenesis stimulation, and regulation of extracellular tissue remodeling [17-19]. Regarding gliomas, microarray gene analyses have revealed that the human YKL-40 gene is one of the most over-expressed genes in GBM, but it is not expressed in normal brain tissue [10,20]. Furthermore, our previous study indicated that silencing the YKL-40 gene could significantly attenuate glioma cell proliferation by arresting the cell cycle in the G_1 _phase [21]. Therefore, YKL-40 is an attractive target in the design of anti-cancer therapies. However, to date, there is no documentation on targeting YKL-40 expression for development of a novel adjuvant glioma therapy.

In 2007, we reported that a polyphenolic natural product, resveratrol (Res), inhibited cell growth and induced apoptosis of C6 glioma cells in rats but not in normal 3T3 fibroblast cells [22]. In *in vivo *studies, Res slowed the growth of subcutaneous gliomas in rats [23], prolonged survival time, and increased the survival rate in a rat glioma model [24]. Thus, Res has significant potential clinical applications in the treatment of gliomas. On the basis of existing literature reports and our previous studies, we hypothesize that Res might repress YKL-40 expression, and the findings of the present study have confirmed this to be the case. To the best of our knowledge, this is the first report indicating that Res could be a potential candidate targeting YKL-40 expression.

## Methods

### Cell lines and culture

A human glioblastoma cell line, U87, was purchased from the American Type Culture Collection (ATCC; Manassas, VA, USA). The tumour cell line was maintained in minimum essential medium (MEM) (Gibco Life Science, Gaithersburg, MD, USA) supplemented with 10% fetal bovine serum (Gibco Life Science), 2 mM L-glutamine, 100 units/ml penicillin, and 100 μg/ml streptomycin. All cells were cultured at 37°C in a 5% CO_2 _incubator.

### Cell viability assay

Cell proliferation was determined by using a WST-1 cell proliferation reagent (Roche Applied Science, Mannheim, Germany). In brief, 2 × 10^4 ^cells were seeded in a flat-bottomed 96-well plate (Nunc, Roskilde, Denmark) and cultured for 24 h to reach 60% to 70% confluence prior to Res treatment. Res (Sigma, St Louis, MO, USA) was dissolved in dimethylsulfoxide (DMSO; Wako, Osaka, Japan) to a stock concentration of 1 mM and subsequently diluted with culture medium to final concentrations of 0, 1, 10, and 100 μM just before use. The concentration of DMSO in the vehicle was 1:1000. The cells were exposed to the indicated concentrations of Res for 0, 24, 48, and 72 h. Each group was set in 8 wells. At each time point, 10 μl/well WST-1 reagent cultured at 37°C for 4 h was added. The optical density was measured at 460 nm. The cell growth curves were drawn according to the optical density of each group of cells. The trypan blue dye cell exclusion assay was used to determine cell viability by treating the cells with different dosages of Res for 48 h. Cell viability is described as a percentage of the DMSO control group, which was set as 100%. All experiments were performed in triplicate.

### Cell transfection and the siRNA method

Cells were seeded at a concentration of 2.5-5 × 10^4 ^cells/ml and cultured for 24 h in 6-well plates in order to reach 60% to 70% confluence before transfection. The cells were then transfected with 20 nM *SMART *pool^® ^*Reagents *YKL-40 or non-targeting (random) siRNA (Dharmacon, Lafayette, CO, USA) in the presence of lipofectamine 2000 (Invitrogen, Carlsbad, CA, USA) according to the manufacturer's instructions. In brief, lipofectamine (Invitrogen) and YKL-40 siRNA or negative control siRNA were dissolved separately in antibiotic- and serum-free MEM. After 10 min of equilibration at room temperature, each RNA solution was combined with the respective volume of lipofectamine solution, mixed gently, and allowed to form siRNA liposomes for 20-30 min at room temperature. After the cells were washed 3 times with PBS, the 200 μl/well siRNA liposome mixture was added followed by the addition of 800 μl/well antibiotic- and serum-free MEM. The cells were incubated for 4 h, and the media were subsequently changed to antibiotic- and serum-containing MEM.

### Cell invasion assay

The cell invasion assay was carried out using modified Boyden chambers consisting of transwell-precoated matrigel membrane filter inserts with 8-μm pores in 24-well tissue culture plates (BD Biosciences, San Diego, CA, USA). U87 cells were pretreated with 100 μM Res or YKL-40 siRNA for 48 h. The cells were then seeded and plated at 5 × 10^4 ^cells/well in the upper transwell chambers of inserts precoated with matrigel in serum- and growth-factor-free medium. The bottoms of the chambers were filled with 500 μl medium containing 10% FBS. After 24 or 48 h, matrigel-precoated inserts were fixed and stained with Diff-Quick Fixative Solutions (Dade Behring, Newark, DE, USA). Attached cells were imaged with an Olympus digital camera mounted to a light microscope and quantified using ImageJ software http://rsb.info.nih.gov/ij/. Invasion was calculated as the ratio of the number of invading cells to the total number of cells in both sides of the membrane. Cells were counted in triplicate membranes.

### Transfection of U87 cells and luciferase reporter gene assay

The YKL-40 promoter was cloned with PCR from human genomic DNA using the following oligonucleotides: sense 5'-TAT CTG GTA CCG TGC AGG AGT GGG AGG AAG G-3' and anti-sense 5'-GAT CTA AGC TTC ATT CTG GCT GCA GCA GAG C-3'. The sequences were obtained from the published human YKL-40 promoter sequence [15]. The resultant 380-bp fragment was inserted into the *Kpn*I and *Hin*dIII sites of the PGV-B2-basic luciferase reporter vector (ToyoInk, Tokyo, Japan), which contains the human YKL-40 gene sequences spanning the region between -377 and 2 bp. Purified reporter plasmids were then transfected into U87 cells via the lipofectamine (Invitrogen, Carlsbad, CA, USA) transfection method. Reporter plasmid-transfected U87 cells were subsequently treated with 100 μM Res for 48 h. All assays were corrected for total amounts of protein per reaction. Transfected cells were harvested in an aliquot of the cytoplasmic fraction. For the luciferase assay, 40 μl aliquots were taken and the assay was carried out according to the manufacturer's instructions (ToyoInk).

### Real-time quantitative reverse transcript-polymerase chain reaction

Total RNA was extracted with Isogen (Nippon Gene, Toyama, Japan), according to the manufacturer's instructions. cDNA was generated with the First-strand cDNA Synthesis Kit (Amersham Biosciences, Piscataway, NJ, USA), according to the manufacturer's instructions. Total RNA (1 μg) was used for each reaction with polyT primers. Quantitative real-time PCR was performed using an ABI Prism 7000 (Applied Biosystems, Foster City, CA, USA) with ABI SYBR Green PCR Master Mix (Applied Biosystems), human GADPH as an internal control, and primers for YKL-40 (sense 5'-CCT GCT CAG CGC AGC ACT GT-3' and antisense 5'-GCT TTT GAC GCT TTC CTG GTC-3').

### ELISA for YKL-40 in the culture medium

The amount of YKL-40 protein secreted by the cells was measured in the collected medium samples with a two-site sandwich-type ELISA (Quidel Corporation, San Diego, CA, USA) according to the manufacturer's instructions. Protein concentrations were determined as the absorbance measured using the Bio-Rad Benchmark Microplate Reader. No detectable YKL-40 was found in MEM containing 10% FBS. Each time point test was analyzed in triplicate.

### Western blotting

U87 cells were treated with Res (100 μM) and 20 μM PD98059 (Calbiochem, San Diego, CA, USA) for 48 h. DMSO (1:1000)-containing media served as vehicle. Western blotting was then performed as described previously [22] with primary antibodies against YKL-40 (R & D Systems, Minneapolis, MN, USA), -actin, Phospho-ERK1/2, and ERK1/2 (Cell Signaling Technology, Beverly, MA, USA), followed by incubation for 1 h at room temperature with a secondary antibody (HRP-conjugated anti-rat or anti-rabbit IgG; Cell Signaling Technology). Immunoreactive bands were visualized using enhanced chemiluminescence (Amersham Pharmacia Biotech, Buckinghamshire, UK) and quantified with LAS-1000 plus (Fujifilm, Tokyo, Japan).

### Statistical analysis

All statistical analyses were performed with the Statistical Package for the Social Sciences (SPSS) 13.0 (SPSS inc., Chicago, IL). Statistical significance between more than 2 groups was determined using one-way ANOVA; statistical significance between 2 groups was determined using Student's *t*-test. The significance level of all tests was set at *P *< 0.05

## Results

### Res-induced inhibition of cellular proliferation in U87 glioblastoma cells

**Figure 1:**
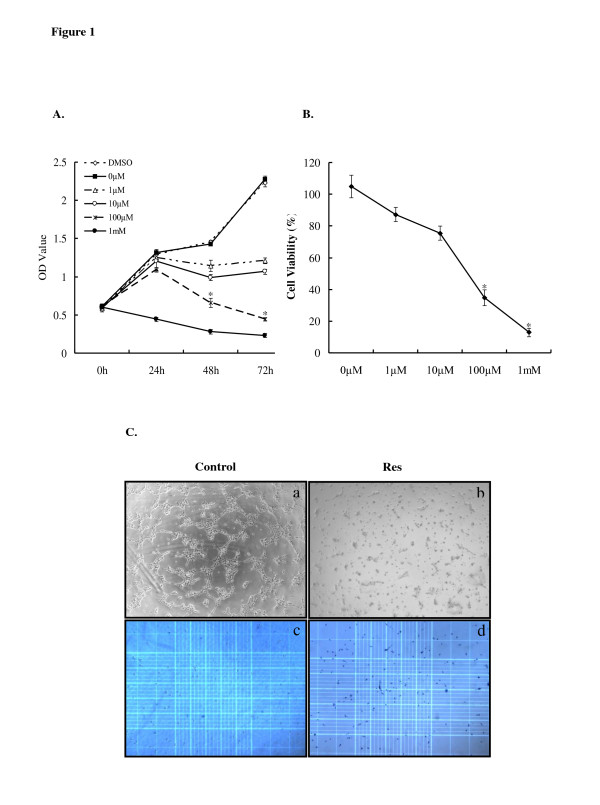
**U87 cell proliferation assay**. **A**. The WST-1 assay was performed to measure U87 cell viability after treatment with increasing concentrations of Res (DEMSO, 0, 1, 10, and 100 μM, and 1 mM) for different exposure times (0, 24, 48, or 72 h). The groups treated with 100 μM Res for 48 and 72 h showed the greatest significant difference compared with the other groups (*P *< 0.01). Asterisks indicate statistically significant changes (*P *< 0.01) after Res treatment. Bars indicate SE. **B**. The trypan blue dye exclusion assay revealed the U87 cell viability after treatment with increasing concentrations of Res (0, 1, 10, and 100 μM, and 1 mM) for 48 h. Asterisks indicate statistically significant changes (*P *< 0.01) after Res treatment. Bars indicate SE. **C**. Morphology of U87 cells treated by 100 μM Res for 48 h under a light microscope (a.b); Morphology of U87 cells treated by 100 μM Res for 48 h in trypan blue dye cell exclusion assay (c.d). Original magnification: 40×.

Aggressive tumour cell proliferation is one of the most pronounced characteristics during the initiation and progression of gliomas. Because of Res' potent cancer chemopreventive effect in carcinogenesis, we first performed cell viability assays with WST-1 reagent. U87 cells were treated with increasing concentrations of Res (DMSO; 0, 1, 10, and 100 μM, and 1 mM) for different exposure times (0, 24, 48, and 72 h) (Figure 1A). In general, the proliferation of U87 cells was inhibited by Res in both a dose- and time-dependent manner. However, 1 mM Res was the maximum dosage for U87 cells. After being treated with 1 mM Res for 24 h, U87 cells exhibited the distinct features of cell death under the light microscope (data not shown). Interestingly, at 72 h, 1 μM Res treatment significantly (*P *< 0.01) inhibited cells compared to treatment with 0 μM. The groups treated with 100 μM Res for 48 and 72 h exhibited the most statistically significant differences compared with those of the other groups (*P *< 0.01) although there was no significant difference between the 2 groups themselves (*P *> 0.05). At 48 h, cell viability was further reinforced by the trypan blue dye exclusion assay (Figure 1B). Cell morphological changes are shown in Figure 1C. Therefore, we chose 100 μM Res for 48 h as the critical point for further study.

### Res-induced decrease of U87 cell invasiveness

**Figure 2:**
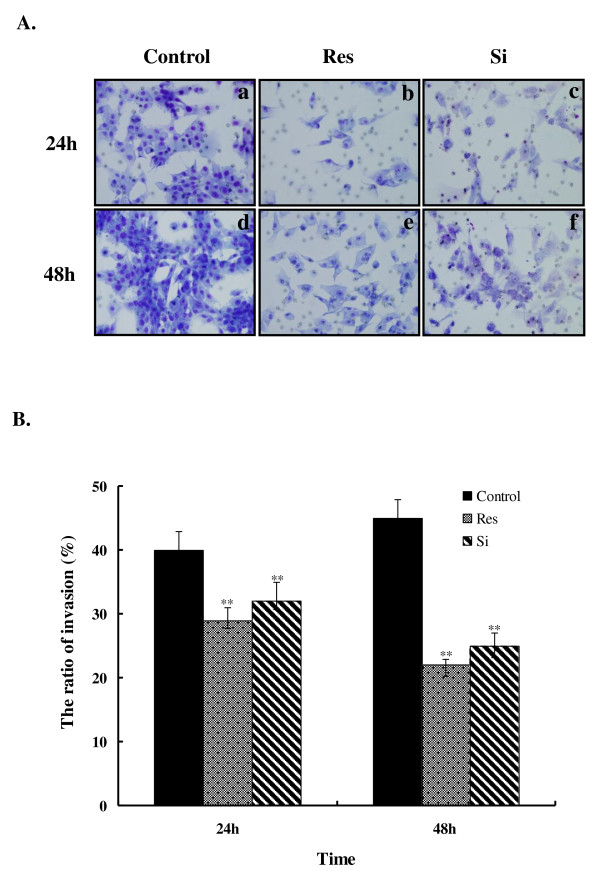
**Matrigel invasion assay of U87 cells pretreated with 100 μM Res and siRNA YKL-40 for 48 h**. **A**. A modified Boyden chamber with a matrigel-precoated membrane filter insert was used to measure *in vitro *invasiveness. After 24 or 48 h of incubation, cells that invaded through the membrane were stained and representative fields were photographed. Original magnification: 200 ×. **B**. Invasion was calculated as the ratio of the number of invading cells to total cell numbers in both sides of the membrane. The ratio of invading cells was significantly reduced by Res treatment. Asterisks indicate statistically significant changes (*P *< 0.01) after Res treatment. Columns represent the average number of invading cells per field of at least 5 fields from 3 independent experiments. Bars indicate SE.

It is well known that the highly invasive characteristics of malignant glioma cells account for the resultant poor therapeutic effects of radical excision. We assessed whether the invasiveness glioma cells can be inhibited by Res. U87 cells were pretreated with 100 μM Res or 20 nM YKL-40 siRNA for 48 h. The same number of cells was seeded, and the invasiveness of the U87 cells was then determined in a modified Boyden chamber with a matrigel-precoated membrane filter insert. After incubation for 24 or 48 h, the ratio of invading cells decreased significantly (*P *< 0.01) (Figure 2) in both Res- and siRNA-pretreated U87 cells.

### Res-induced repression of YKL-40 promoter activity, mRNA transcription, and protein expression

**Figure 3:**
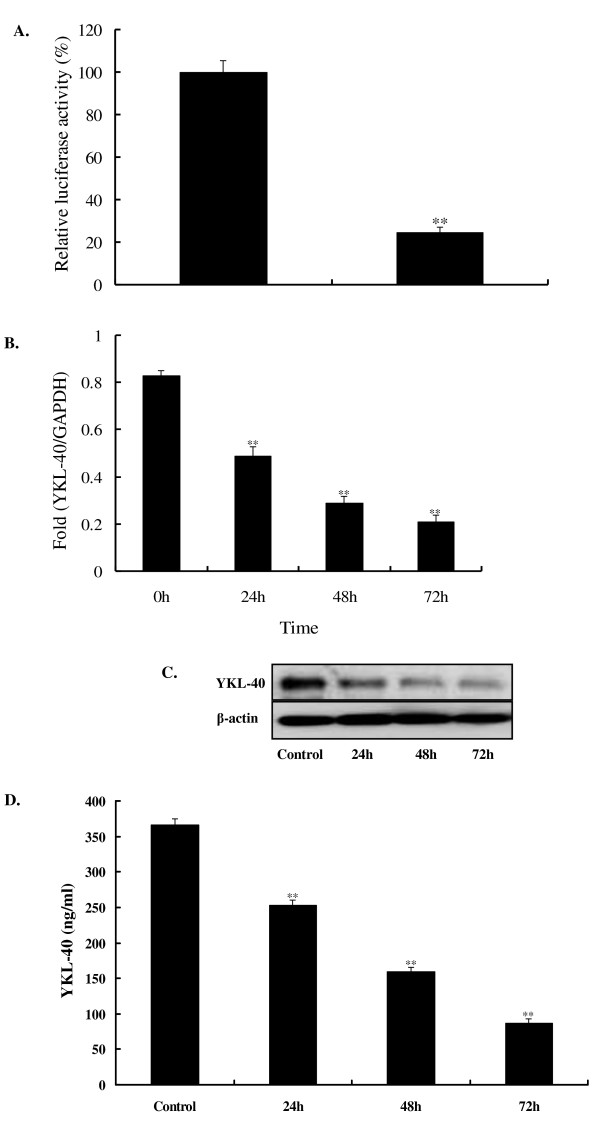
**Res repressed YKL-40 promoter activity, mRNA transcription, and protein expression**. **A**. Luciferase assay after YKL-40 promoter reporter plasmid-transfected U87 cells after treatment with 100 μM Res for 48 h. **B**. YKL-40 mRNA transcription level measured using real-time RT-PCR after treatment with 100 μM Res for 24, 48, and 72 h. **C**. YKL-40 protein expression levels measured using western blotting after treatment with 100 μM Res for 24, 48, and 72 h. **D**. Secreted YKL-40 protein levels in media measured using ELISA after treatment with 100 μM Res for 24, 48, or 72 h. Asterisks indicate statistically significant changes (*P *< 0.01). Bars indicate SE.

In our previous study, the silencing of YKL-40 inhibited the proliferation of U87 cells [21]. In conjunction with the cellular proliferation and invasion induced by Res *in vitro*, we further speculated that Res could decrease the expression of YKL-40. To test this hypothesis, YKL-40 promoter was synthesized, and the luciferase assay was conducted. Real-time RT-PCR, western blotting, and ELISA were used to evaluate the mRNA transcription and protein expression levels. After treatment with 100 μM Res for 48 h, YKL-40 promoter activity was significantly reduced compared with that of the control group (*P *< 0.01) (Figure 3A). YKL-40 mRNA transcription and protein expression levels were consistently and steadily repressed in a time-dependent manner (Figure 3B-D). Thus, Res repressed YKL-40 in U87 cells by decreasing the activity of its promoter, mRNA transcription, and protein expression levels *in vitro*.

### Involvement of the ERK1/2 pathway in the Res-induced repression of YKL-40 expression

**Figure 4:**
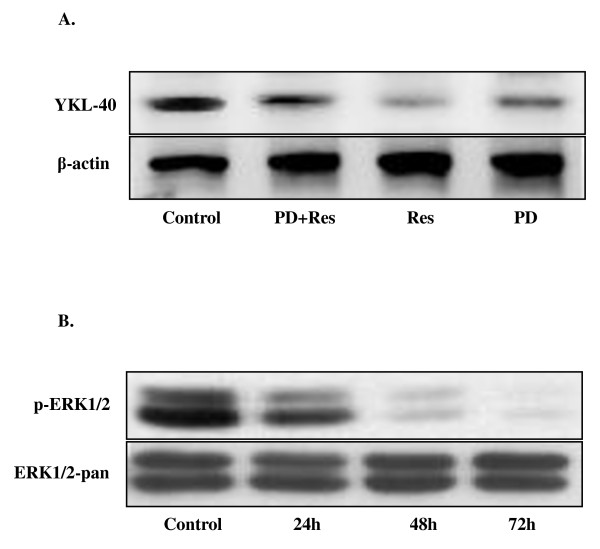
**ERK1/2 pathway involvement in the Res-induced repression of YKL-40 expression**. **A**. YKL-40 protein expression after treatment with 20 nM PD98059 alone, 20 nM PD98059 along with 100 μM Res, or with 100 μM Res alone for 48 h by western blotting. **B**. Phospho-ERK1/2 expression after treatment with 100 μM Res for 24, 48, and 72 h determined by western blotting. Asterisks indicate statistically significant changes (*P *< 0.01). Bars indicate SE.

Extracellular regulated kinase 1/2 (ERK1/2) is well known for its critical role in cell proliferation. In this study, we hypothesized that the ERK1/2 pathway is involved in the down-regulation of YKL-40 expression by Res. In order to demonstrate this, the specific inhibitor of the ERK1/2 pathway, PD98059 (20 μM), was added along with 100 μM Res to the U87 cell culture for 48 h. The band densities of western blotting revealed that YKL-40 protein expression decreased most significantly in the Res-treated group (*P *< 0.01); in addition, PD98059 partially blocked the expression of YKL-40 (Figure 4A). Moreover, phosphor-ERK1/2 expression was reduced by Res treatment in a time-dependent manner (Figure 4B).

### Discussion

The prominent hallmarks of gliomas are their characteristics of malignant proliferation and aggressive invasion. In a pioneering study, Nigro and colleagues determined that immortalized human astrocytes stably transfected with YKL-40 exhibited changes in gene expression similar to those observed in human tumours and confer radioresistance and an increased invasive capacity *in vitro *[17]. Our previous study showed that the YKL-40 expression level was well correlated to the pathological grade of human primary astrocytomas and acted as a proliferation and anti-apoptosis factor in astrocytoma cells [21]. These findings suggest that YKL-40 is a positive regulator in the proliferation and invasiveness of glioma cells. In the present study, we again determined that the invasion ability of U87 cells is sharply impaired after YKL-40 gene silencing. The numerous literary reviews and our accumulated findings suggest that YKL-40 is an attractive target for selective anticancer drug development. Our study reveals for the first time that a drug - Res - repressed the expression of YKL-40 by decreasing its promoter activity, mRNA transcription, and protein expression levels in U87 cells *in vitro*.

It is widely known that Res is synthesized by a wide range of plant species, including grapes, peanuts, pines, and more than 70 other species in response to injury, ultraviolet irradiation, or fungal attack. In particular, Res is abundant in the skin of red grapes and may account for the "French Paradox" [25]. Due to its various beneficial health effects, such as its anti-oxidant, anti-inflammatory, and cardioprotective activities, Res is considered as a "state-of-the-art" medicine derived from natural sources [26-28]. However, it was not until 1997 when Jang and colleagues [25] published a seminal article demonstrating the anti-carcinogenic effects of Res did this phytoalexin attract considerable attention from oncologists as a novel chemopreventive agent. Shortly after, tremendous progress was made in revealing the potential role of Res in the anti-carcinogenic process. Over the past decades, numerous preclinical findings indicate that Res inhibits the proliferation and induces apoptosis of various cancer cells cultured *in vitro *in addition to retarding the growth of implanted tumours *in vivo *[22-28]; this suggests the existence of an inverse relationship between Res and its striking inhibition associated with tumour initiation, promotion, and progression. Recent studies have also revealed that the chemopreventive effect of Res is significantly stronger than that of cisplatin (CP); furthermore, co-administration of Res (20 mg/kg) markedly enhances the chemoprevention of CP in the Ehrlich ascites carcinoma (EAC) solid tumour model [29]. Our current study further confirms that Res inhibits the proliferation of U87 cells in both a dose- and time-dependent manner. More interestingly, we demonstrated that Res significantly decreased the ability of U87 cells to invade *in vitro*. Thus, Res has promising roles in natural cancer prevention and treatment [30].

As the most distinct member of the mitogen-activated protein kinase (MAPK) family, the ERK pathway is synonymous with cell proliferation. YKL-40 expression is positively associated with the expression of phosphor-ERK1/2, which is strongly correlated with poor response to radiotherapy and negative outcomes [31]. In addition, phosphor-ERK1/2 expression is notably decreased in U87 cells in which YKL-40 is silenced [21]. Therefore, we speculate that the ERK1/2 pathway is involved in the down-regulation of YKL-40 induced by Res. The ERK1/2-specific inhibitor, PD98059 (20 μM), was added along with 100 μM Res to the culture medium and the U87 cells were cultured for 48 h. As expected, YKL-40 expression decreased most significantly in the Res-treated group and partially decreased in PD98059-treated group. This indicates that the ERK1/2 pathway is involved in Res repressed YKL-40 expression in U87 cells. Moreover, the functional impact of Res on MAPK signaling may depend, to some extent, on the concentrations of Res used under different conditions and on the biological systems involved. Relatively low concentrations of Res (1 pM to 10 μM) are reported to induce phosphor-ERK1/2 in human neuroblastoma cells, whereas higher concentrations (50 to 100 μM) negatively interfere with MAPK phosphorylation [32]. In human coronary smooth muscle cells (HCSMC), pretreatment with Res (1-100 μM) triggered marked inhibition of endothelin-1-evoked cell proliferation and ERK1/2 activation [33]. In the present study, we consistently used 100 μM Res and observed significantly decreased phosphor-ERK1/2 activity in a time-dependent manner.

## Conclusion

Our study demonstrates for the first time that Res represses YKL-40 expression by decreasing its promoter activity, mRNA transcription, and protein expression levels in U87 cells *in vitro*; in addition, the ERK1/2 pathway is involved in this process. This finding supports the idea of targeting YKL-40 as a novel adjuvant therapy in glioma treatment and expands the prospective use of Res in anti-glioma research. We hope that any drug that specifically inhibits the expression of YKL-40 might enhance the effects of tumour excision of and improve the prognosis for glioma patients.

## Abbreviations

MAPK: mitogen-activated protein kinase; ERK1/2: extracellular regulated kinase 1/2; Res: resveratrol; ELISA: enzyme linked immunosorbent assay; MEM: minimum essential medium; siRNA: short interference RNA

## Competing interests

The authors declare that they have no competing interests.

## Authors' contributions

WZ, KMu, and XZ designed and performed the study. KMa synthesized the YKL-40 promoter. SD performed real-time RT-PCR. MO, KMi, and NK were responsible for protein analysis. ZF and TT were responsible for the preparation of the manuscript. All authors read and approved the final manuscript.

## Pre-publication history

The pre-publication history for this paper can be accessed here:

http://www.biomedcentral.com/1471-2407/10/593/prepub
